# Fast generation of three-atom singlet state by transitionless quantum driving

**DOI:** 10.1038/srep22202

**Published:** 2016-03-02

**Authors:** Zhen Chen, Ye-Hong Chen, Yan Xia, Jie Song, Bi-Hua Huang

**Affiliations:** 1 Department of Physics, Fuzhou University, Fuzhou 350002, China; 2 Department of Physics, Harbin Institute of Technology, Harbin 150001, China

## Abstract

Motivated by “transitionless quantum driving”, we construct shortcuts to adiabatic passage in a three-atom system to create a singlet state with the help of quantum zeno dynamics and non-resonant lasers. The influence of various decoherence processes is discussed by numerical simulation and the results reveal that the scheme is fast and robust against decoherence and operational imperfection. We also investigate how to select the experimental parameters to control the cavity dissipation and atomic spontaneous emission which will have an application value in experiment.

Quantum entanglement is an intriguing property of composite systems. The generation of entangled states for two or more particles is not only fundamental for demonstrating quantum nonlocality^1^,^2^, but also useful in quantum information processing (QIP)^3^,^4^,^5^,^6^, typically the Bell state, the Greenberger-Horne-Zeilinger (GHZ) state and the W state^7^,^8^,^9^,^10^,^11^,^12^,^13^. Different entangled state has different advantages. For example, the W state is likely to retain bipartite entanglement when any one of the three qubits is traced out, thus it is robust against qubit loss. The GHZ state is the most entangled state and can maximally violate the Bell inequalities^7^. Recently, some attention has been paid to a special type of entangled state called the *N*-particle (*N* ≥ 2) *N*-level singlet state^14^. The form of the *N*-atom singlet state can be expressed as


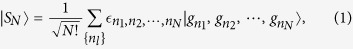


where 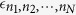 are the generalized Levi-Civita symbols, {*n*_*l*_} are the permutations, and 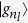 denote the bases of the qubits^15^. It has been shown that the singlet state not only is in connection with violations of Bell inequalities^16^, but also can be used to construct decoherence-free subspace, which is robust against collective decoherence^17^. Moreover, the singlet state can be used to solve several problems which have no classical solutions, including “*N* strangers”, “secret sharing”, “liar detection”, and so on^14^,^17^. Furthermore, the singlet state also can be used in a scheme designed to probe a quantum gate that can realize an unknown unitary transformation^18^. In recent years, lots of theoretical schemes have been proposed to generate singlet state in the cavity quantum electrodynamics (C-QED) system via different techniques^17^,^18^,^19^,^20^,^21^,^22^,^23^. Among these techniques^17^,^18^,^19^,^20^,^21^,^22^,^23^, there are two famous techniques for their robustness against decoherence in proper conditions. One is stimulated Raman adiabatic passage (STIRAP)^20^,^21^, the other is Quantum Zeno dynamics (QZD)^15^,^22^,^23^. In general, adiabatic passage technique has been widely used and an advantage of the technique is that can reduce populations of the intermediate excited states. Therefore, the technique would restrain the influence of atomic spontaneous emission on the fidelity. As we know, the adiabatic condition is managed to be slow to make sure each of the eigenstates of the system evolves along itself all the time without transition to other ones. So, the operation time is long in previous schemes^20^,^21^ via adiabatic passage. Differ from the adiabatic passage, QZD is usually robust against photon leakage but sensitive to atomic spontaneous emission^15^,^22^,^23^. Therefore, some of the researchers introduce detuning between the atomic transition to restrain the influence of atomic spontaneous emission. However, that also increases the operation time. In general, the interaction time for a method is the shorter the better. Otherwise, the method may be useless because the dissipation caused by decoherence, noise, and losses on the target state increases with the increasing of the interaction time^24^.

In order to solve this problem, in recent years researchers pay more attention to “shortcuts to adiabatic passage (STAP)”^25^,^26^,^27^,^28^ which employs a set of techniques to speed up a slow quantum adiabatic process through a non-adiabatic route. Usually STAP can overcome the harmful effect caused by decoherence, noise and losses during the long operation time. Recently, STAP has been applied in a wide range of system to implement quantum information processing (QIP) in theory and experiment^25^,^26^,^27^,^28^,^29^,^30^,^31^,^32^,^33^,^34^,^35^,^36^,^37^,^38^,^39^,^40^,^41^,^42^,^43^,^44^,^45^,^46^,^47^,^48^,^49^,^50^,^51^,^52^,^53^,^54^,^55^,^56^,^57^. In order to construct STAP to speed up adiabatic processes effectively, many methods^25^,^26^,^27^,^28^,^29^,^30^,^31^,^32^,^33^,^34^,^35^,^36^,^37^,^38^,^39^,^40^,^41^ are related. Such as, invariant-based inverse engineering proposed by Muga and Chen^25^,^26^,^27^,^28^,^29^,^30^,^31^, can achieve the fast population transfer within two internal states of a single Λ-type atom. “Transitionless quantum driving” (TQD)^32^,^33^,^34^,^35^ proposed by Berry, provides a very effective method to construct the “counter-diabatic driving” (CDD) Hamiltonian *H*(*t*) which can accurately derive the instantaneous eigenstates of *H*_0_(*t*) to speed up adiabatic processes effectively. But it is also found that the designed CDD Hamiltonian is hard to be directly implemented in practice, especially in multiparticle system. In order to solve the problem, many schemes^29^,^33^,^34^,^45^,^46^ have been put forward. In 2014, by using second-order perturbation approximation twice under large detuning condition and transitionless quantum driving, Lu *et al.* have proposed an effective scheme^45^ to implement the fast populations transfer and prepare a fast maximum entanglement between two atoms in a cavity. The idea inspires that using some traditional methods to approximate a complicated Hamiltonian into an effective and simple one first, then constructing shortcuts for the effective Hamiltonian might be a promising method to speed up evolution process of a system. Later, Chen *et al.*^46^ have proposed a promising method to construct STAP for a three-atom system to generate GHZ states in the cavity QED system in light of QZD and TQD. Their schemes might be useful to realize fast and noise-resistant quantum information processing for multi-qubit system in current technology.

In this paper inspired by the schemes^45^,^46^, we discuss how to construct STAP to fastly generate a three-atom singlet state in cavity QED system by using the approach of “transitionless tracking algorithm”. Based on quantum Zeno dynamics^58^,^59^ and large detuning conditon, we can simplify the original Hamiltonian of system and obtain the effective Hamiltonian equivalent to the corresponding CDD Hamiltonian, the evolution process of system can be speeded up, and the STAP can be achieved in experiment easily. What’s more, numerical investigation shows that our scheme is also fast and robust against both cavity decay and atomic spontaneous emission for three-atom singlet state preparation. It will be much useful in dealing with the fast and noise-resistant generation of *N*-atom singlet state.

The paper is organized as follows. In section II, we describe a theoretical model for three atoms which are trapped in a bimodal-mode cavity. In section III, we demonstrate how to construct STAP for the system in section II, and use the constructed shortcut to generate a three-atom singlet state. The numerical simulation and experimental discussion about the validity of the scheme are also given. Finally, a summary is given in section IV.

## Theoretical Model

**Figure 1:**
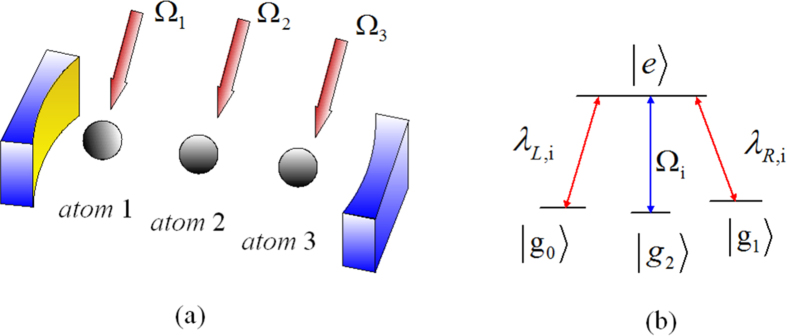
(**a**) Cavity-atom combined system of the three-atom singlet state generation. (**b**) Atomic level configuration for the original Hamiltonian.

The sketch of the experimental setup is shown in Fig. 1. Three identical four-level atoms with three ground states 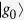, 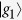 and 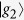, and an excited state 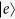 are trapped in a bimodal-mode cavity. The atomic transition 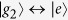 is driven resonantly through classical laser field with time-dependent Rabi frequency Ω(*t*), the transition 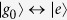 is coupled resonantly to the left-circularly polarized mode of the cavity with coupling *λ*_*L*_, and transition 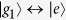 is coupled resonantly to the right-circularly polarized mode of the cavity with coupling *λ*_*R*_. Under the rotating-wave approximation (RWA), the interaction Hamiltonian for this system reads (*ħ* = 1):


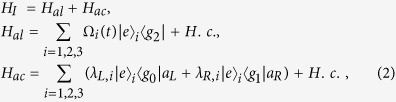


where *a*_*L*_ and *a*_*R*_ are the left-circularly and the right-circularly annihilation operators for cavity mode, respectively. We set *λ*_*L*,*i*_ = *λ*_*R*,*i*_ = *λ* for simplicity. If we assume the initial state of the system is 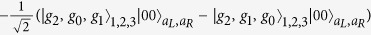, the system will evolve within a single-excitation subspace with basis states


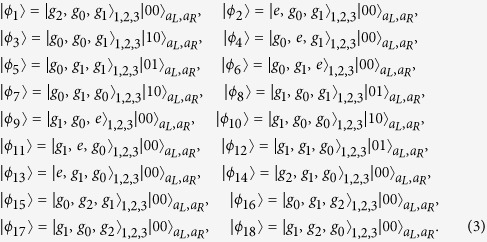


Then, we rewrite the Hamiltonian *H*_*ac*_ and *H*_*al*_ with the eigenvectors of *H*_*ac*_:


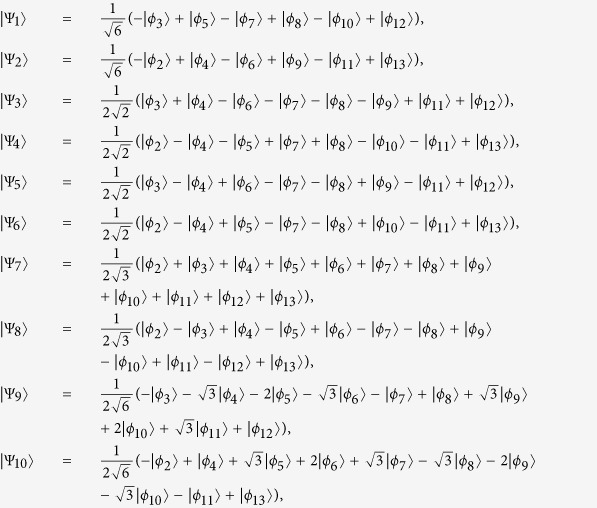



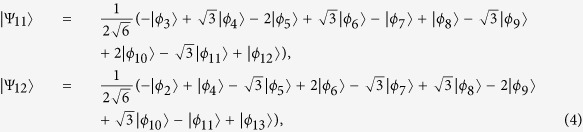


with eigenvalues *η*_1_ = *η*_2_ = 0, *η*_3_ = *η*_4_ = *λ*, *η*_5_ = *η*_6_ = −*λ*, *η*_7_ = 2*λ*, *η*_8_ = −2*λ*, 
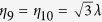
, and 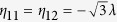. We obtain


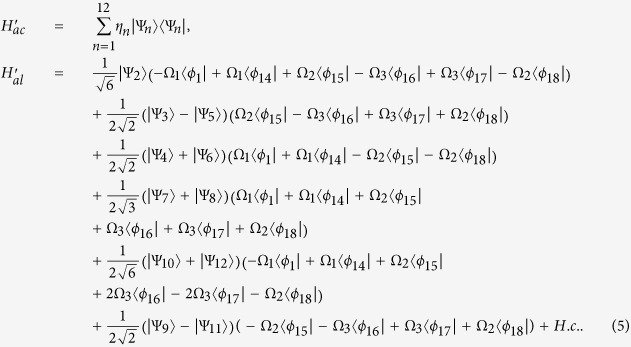


Through performing the unitary transformation 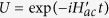 and neglecting the terms with high oscillating frequency by setting the condition Ω_*i*_ ≪ *λ*, we obtain an effective Hamiltonian


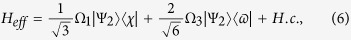


here we set Ω_2_ = Ω_3_, 
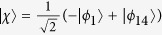
 and 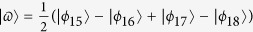.

We can see Hamiltonian in equation (6) as a simple three-level system with an excited state 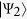 and two ground states 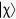 and 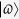. For this effective Hamiltonian, its eigenstates are easily obtained


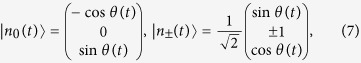


corresponding eigenvalues *ε*_0_ = 0, 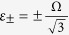, respectively, where 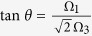 and 
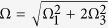
. When the adiabatic condition 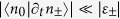 is fulfilled, the initial state 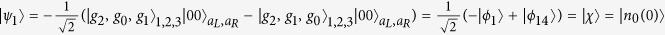 will follow 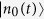 closely, and when 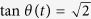, we can obtain the three-atom singlet state:


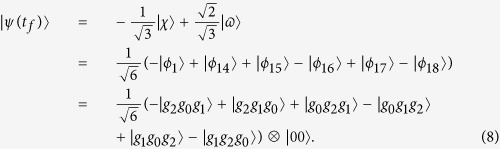


However, this process will take quite a long time to obtain the target state, which is undesirable. We will talk in later.

### Using STAP to generate a three-atom singlet state

The instantaneous eigenstates 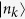 (*k* = 0, ±) for the effective Hamiltonian *H*_*eff*_(*t*) in equation (6) do not satisfy the Schrodinger equation 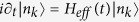. According to Berry’s transitionless tracking algorithm^32^, from *H*_*eff*_(*t*), one can reverse engineer *H*(*t*) which is related to the original Hamiltonian *H*_*I*_(*t*) and can drive the eigenstates exactly. From refs 45, 52, 53, we learn the simplest Hamiltonian *H*(*t*) is derived in the form


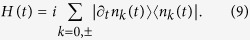


**Figure 2:**
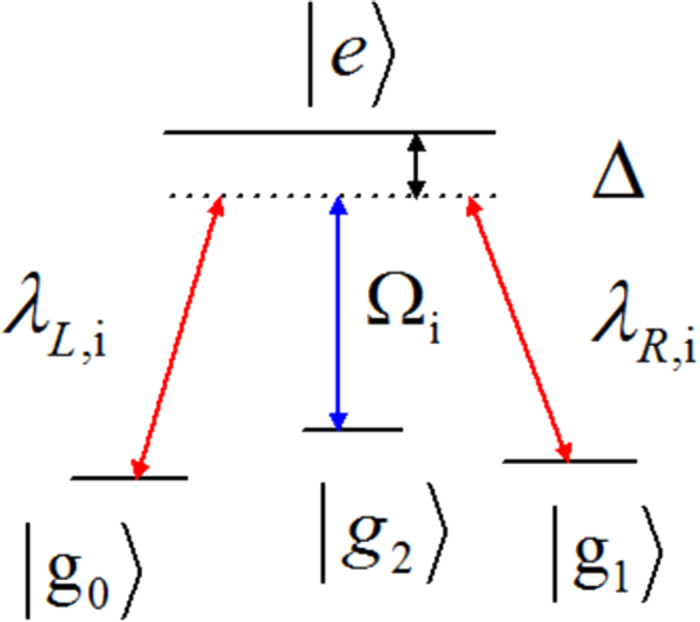
The atomic level configuration for the APF Hamiltonian.

Substituting equation (7) into equation (9), we obtain


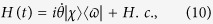


where 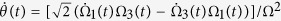. For this three-atom system, the Hamiltonian *H*(*t*) is hard or even impossible to be implemented in real experiment^45^. We should find an alternative physically feasible (APF) Hamiltonian whose effect is equivalent to *H*(*t*). Therefore, we consider that the three atoms are trapped in a cavity and the atomic level configuration is shown in Fig. 2. We make all the resonant atomic transitions into non-resonant atomic transitions with detuning Δ. The non-resonant Hamiltonian reads


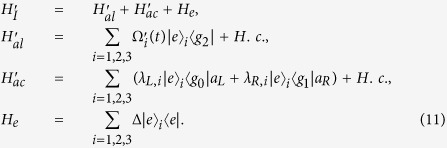


Then similar to the approximation for the Hamiltonian from equation (2) to equation (6) in section II, we also obtain an effective Hamiltonian for the present non-resonant system^15^


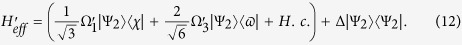


By adiabatically eliminating the state 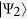 under the condition 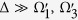, we obtain the final effective Hamiltonian


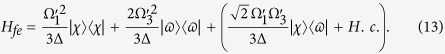


When we choose 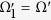 and 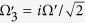 (here Ω′ is a real number), the first two terms can be removed, and the Hamiltonian in equation (13) becomes


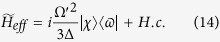


That means, as long as 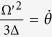, 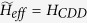, the Hamiltonian for speeding up the adiabatic dark state evolution governed by 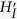 under condition 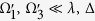 has been constructed. Hence, Ω′ is given


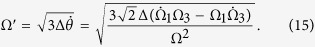


**Figure 3:**
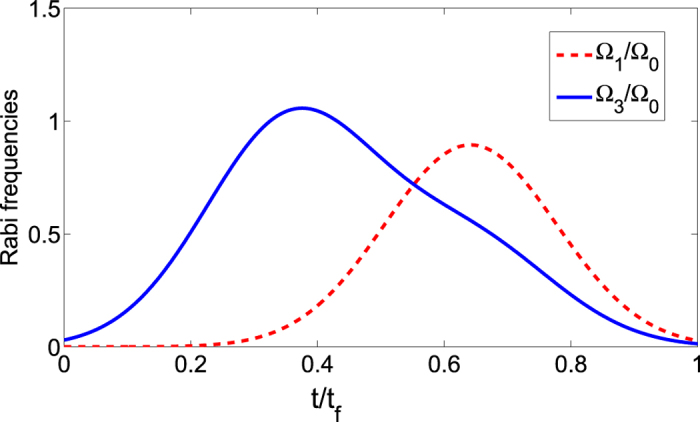
Dependence on *t*/*t*_*f*_ of Ω_1_/Ω_0_ and Ω_3_/Ω_0_.

We will show the numerical analysis of the creation of a three-atom singlet state governed by 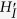. To satisfy the boundary condition of the fractional stimulated Raman adiabatic passage (STIRAP),


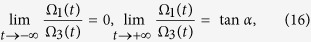


the Rabi frequencies Ω_1_(*t*) and Ω_3_(*t*) in the original Hamiltonian *H*_*I*_(*t*) are chosen as


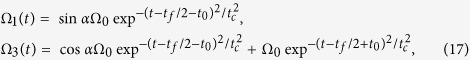


where Ω_0_ is the pulse amplitude, *t*_*f*_ is the operation time, and *t*_0_, *t*_*c*_ are some related parameters. In order to create a three-atom singlet state, the finial state 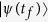 should be 
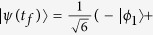

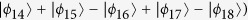
 according to equation (8). Therefore, we have tan *α* = 2. And choosing parameters for the laser pulses suitably to fulfill the boundary condition in equation (16), the time-dependent Ω_1_(*t*) and Ω_3_(*t*) are gotten as shown in Fig. 3 with parameters *t*_0_ = 0.14*t*_*f*_ and *t*_*c*_ = 0.19*t*_*f*_.

**Figure 4:**
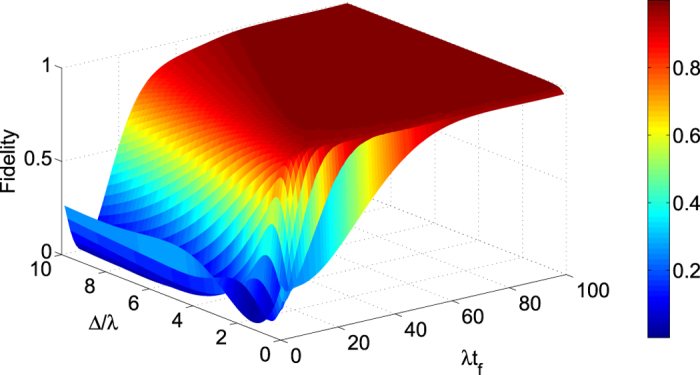
The fidelity *F* of the three-atom singlet state versus the interaction time *λt*_*f*_ and the detuning Δ/*λ*.

Figure 4 shows the relationship between the fidelity of the generated three-atom singlet state (governed by the APF Hamiltonian 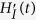) and two parameters Δ and *t*_*f*_ when Ω_0_ = 0.2*λ*, where the fidelity for the three-atom singlet state is given through 
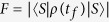
 (*ρ*(*t*_*f*_) is the density operator of the whole system when *t* = *t*_*f*_). It’s easy to find that there is a wide range of selectable values for parameters Δ and *t*_*f*_ to get a high fidelity. And the fidelity increases with the increasing of *t*_*f*_ while decreases with the increasing of Δ. This is easy to understand. If we set 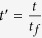, according to equation (17), we can obtain two dimensionless parameters


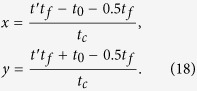


Therefore, putting equations (17) and (18) into equation (15), we obtain


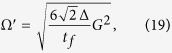


where


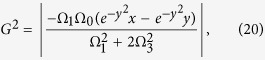


is a dimensionless function. A brief analysis of *G* tells that the amplitude of *G* is close to 1. That is, the amplitude of Ω′ is mainly dominated by 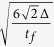. In order to satisfy the condition Ω′ ≪ *λ* and Ω′ ≪ Δ, we can work out Δ/*t*_*f*_ ≪ 1 and Δ*t*_*f*_ ≫ 1. So, long *t*_*f*_ can lead to a high fidelity as shown in Fig. 4. When the detuning Δ is smaller or near 0, it’s not meet the condition Δ*t*_*f*_ ≫ 1, so the fidelity is lower in a short time as shown in Fig. 4. We know 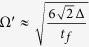, shortening the evolution time implies that relative large laser intensities is required, and this would destroy the Zeno condition. Yet slightly destroying the Zeno condition is also helpful to achieve the target state in a much shorter interaction time^45^,^47^.

**Figure 5:**
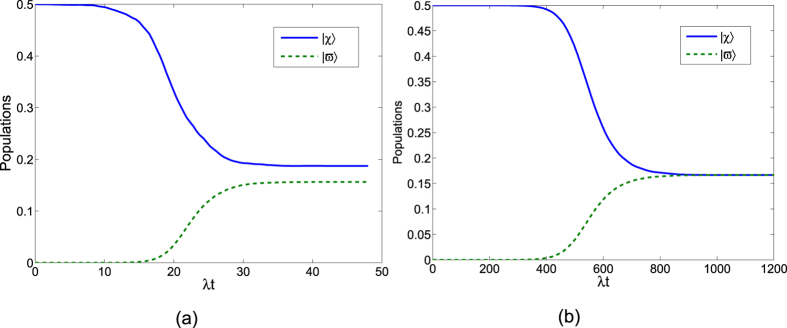
(**a**) Time evolution of the populations for the states 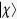 and 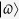 with Ω_0_ = 0.2*λ*, *t*_*f*_ = 40/*λ* and Δ = 3*λ* governed by the APF Hamiltonian 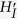. (**b**) Time evolution of the populations for the states 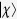 and 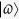 with Ω_0_ = 0.2*λ* and *t*_*f*_ = 1000/*λ* governed by the original Hamiltonian *H*_*I*_.

**Figure 6:**
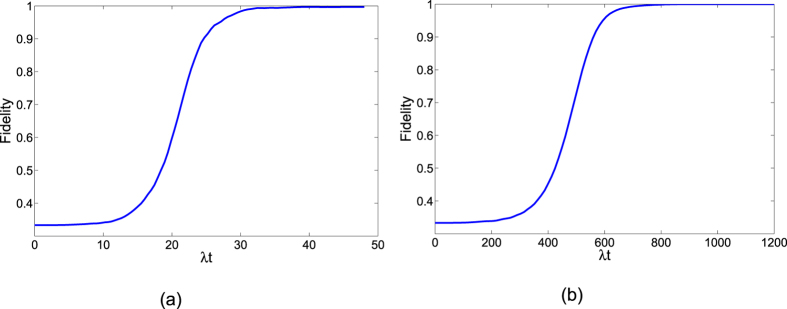
(**a**) The fidelity of the three-atom single state governed by 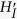. (**b**) The fidelity of the three-atom single state governed by *H*_*I*_.

Next, to comfirm the operation time required for the creation of the three-atom singlet state governed by 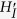 is much shorter than that governed by *H*_*I*_, we contrast the performances of population transfer from the initial state 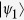 in Fig. 5. The time-dependent population for any state 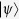 is given by 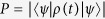, where *ρ*(*t*) is the corresponding time-dependent density operator. Figure 5(a) shows time evolution of the populations for the states 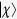 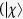 is the initial state 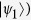 and 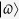 governed by the APF Hamiltonian 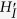 with Ω_0_ = 0.2*λ*, *t*_*f*_ = 40/*λ* and Δ = 3*λ*. Figure 5(b) shows time evolution of the populations for the states 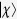 and 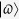 governed by the original Hamiltonian *H*_*I*_ with Ω_0_ = 0.2*λ* and *t*_*f*_ = 1000/*λ*. The comparison of Fig. 5(a,b) shows that with this set of parameters, the APF Hamiltonian 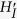 can govern the evolution to achieve a near-perfect three-atom singlet state from state 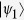 in short interaction time while the original Hamiltonian *H*_*I*_ can not. We also plot the fidelities of the evolved states governed by 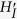 and *H*_*I*_ in Fig. 6, with respect to the target three-atom singlet state. As shown in Fig. 6, when the interaction time *t*_*f*_ = 40/*λ*, the fidelity governed by 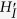 is already 99.98%. While, when *t*_*f*_ = 1000/*λ*, the fidelity governed by *H*_*I*_ achieves 99.93%. The interaction time required for creation of the three-atom singlet state via STAP is much shorter than adiabatic passage.

**Figure 7:**
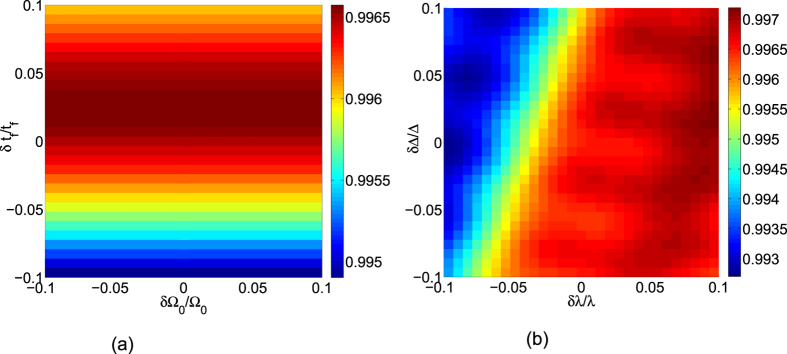
(**a**) The fidelity of the three-atom singlet state versus the variations of total operation time *t*_*f*_ and laser amplitude Ω_0_. (**b**) The fidelity of the three-atom singlet state versus the variations of coupling constant *λ* and the detuning Δ.

**Figure 8:**
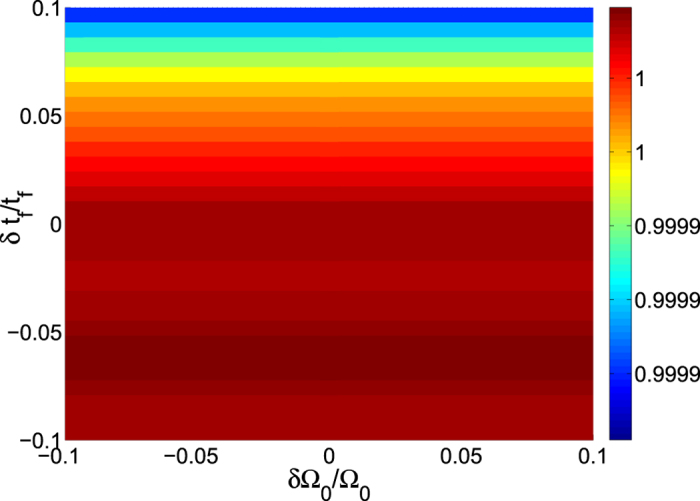
The influence of fluctuations versus total operation time *t*_*f*_ and laser amplitude Ω_0_ on the fidelity for the STIRAP.

Since most of the parameters are hard to faultlessly achieve in experiment, we need to investigate the variations in the parameters induced by the experimental imperfection. We calculate the fidelity by varying error parameters of the mismatch between the laser amplitude Ω_0_ and the total operation time *t*_*f*_, the detuning Δ and the cavity mode with coupling constant *λ*, respectively. We define *δx* = *x*′ − *x* as the deviation of *x*, here *x* denotes the ideal value and *x*′ denotes the actual value. Then in Fig. 7(a) we plot the fidelity of the three-atom singlet state versus the variations in total operation time *t*_*f*_ and laser amplitude Ω_0_. In Fig. 7(b) we plot the fidelity of the three-atom singlet state versus the variations in coupling constant *λ* and the detuning Δ. We find that the scheme is robust against all of these variations. For example, a deviation *δ*Δ/Δ = 10% and *δλ*/*λ* = −10% only causes a reduction about 0.66% in the fidelity. In order to have a fair comparison, we show the influence of fluctuations versus total operation time *t*_*f*_ and laser amplitude Ω_0_ on the fidelity for the STIRAP in Fig. 8. As we can find, the STIRAP scheme almost perfectly restrain the influence caused by the parameters’ fluctuations without doubt. Nevertheless, in Fig. 7(a) we can find that the fidelity of the target state for the STAP is still higher than 99.5% even when the deviation *δ*Ω_0_/Ω_0_ = *δt*_*f*_/*t*_*f*_ = 10%, so we can say the scheme via STAP is also robust against these variations.

**Figure 9:**
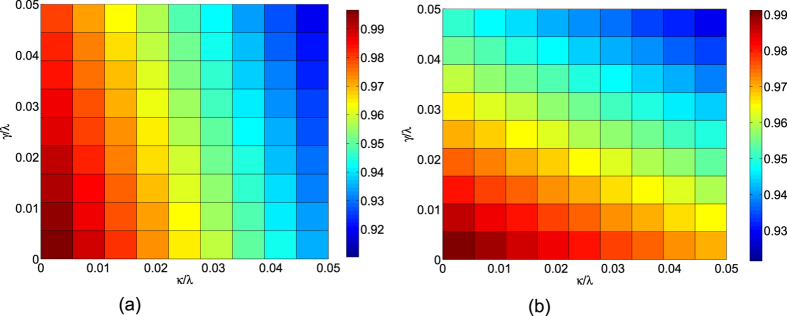
(**a**) The fidelity of the three-atom singlet state governed by the APF Hamiltonian 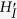 versus *κ*/*λ* and *γ*/*λ* with Ω_0_ = 0.2*λ*, Δ = 3*λ* and *t*_*f*_ = 40/*λ*. (**b**) The fidelity of the three-atom singlet state governed by the APF Hamiltonian 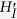 versus *κ*/*λ* and *γ*/*λ* with Ω_0_ = 0.2*λ*, Δ = *λ* and *t*_*f*_ = 40/*λ*.

**Figure 10:**
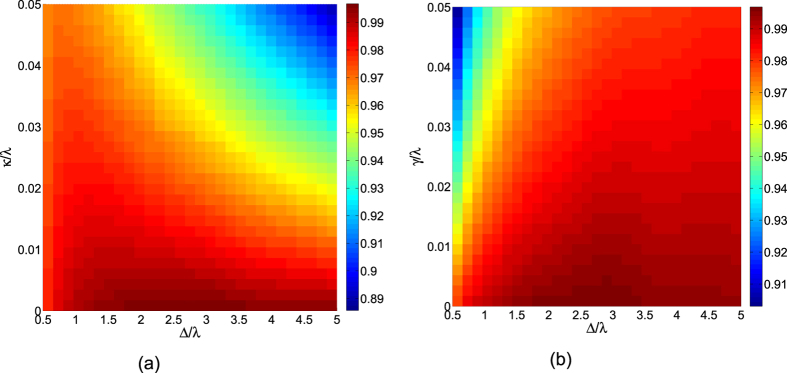
(**a**) The fidelity of the three-atom singlet state versus *κ*/*λ* and Δ/*λ* with Ω_0_ = 0.2*λ*, *t*_*f*_ = 40/*λ*, and *γ*/*λ* = 0. (**b**) The fidelity of the three-atom singlet state versus *γ*/*λ* and Δ/*λ* with Ω_0_ = 0.2*λ*, *t*_*f*_ = 40/*λ*, and *κ*/*λ* = 0.

Next, we will analyze the influence of dissipation induced by the atomic spontaneous emission and the cavity decay. The master equation of motion for the density matrix of the whole system can be expressed as


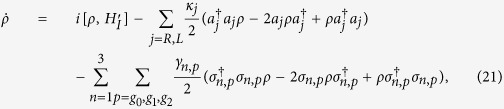


where *ρ* is the density operator for the whole system, *γ*_*n*,*p*_ is the spontaneous emission rate from the excited state 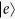 to the ground states 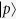 (*p* = *g*_0_, *g*_1_, *g*_2_) of the *n*th (*n* = 1, 2, 3) atom. *κ*_*L*_ (*κ*_*R*_) is the decay rate of the left(right)-circular cavity mode. For simplicity, we assume *κ*_*L*_ = *κ*_*R*_ = *κ* and *γ*_*n*,*p*_ = *γ*. Figure 9(a,b) show the fidelities of the three-atom singlet state governed by the APF Hamiltonian 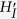 versus *κ*/*λ* and *γ*/*λ* with {Ω_0_ = 0.2*λ*, Δ = 3*λ*, *t*_*f*_ = 40/*λ*} and {Ω_0_ = 0.2*λ*, Δ = *λ* and *t*_*f*_ = 40/*λ*}, respectively. We can find the fidelity *F* decrease slowly with the increasing of cavity decay and atomic spontaneous emission. When *κ* = *γ* = 0.05*λ*, we still can create a three-atom singlet state with a high fidelity 91.03% as shown in Fig. 9(a). By comparing Fig. 9(a,b), we find the effect of the atomic spontaneous emission and cavity field dissipation varies with different parameters values. So, we plot the fidelity of the three-atom singlet state versus *κ*/*λ* and Δ/*λ* with Ω_0_ = 0.2*λ*, *t*_*f*_ = 40/*λ*, and *γ*/*λ* = 0 in Fig. 10(a). Figure 10(b) shows the fidelity of the three-atom singlet state versus *γ*/*λ* and Δ/*λ* with Ω_0_ = 0.2*λ*, *t*_*f*_ = 40/*λ*, and *κ*/*λ* = 0. We find that when *κ*/*λ* is nonzero, the fidelity *F* decreases with the increasing of Δ/*λ* as shown in Fig. 10(a). When *γ*/*λ* is nonzero, the fidelity *F* increases with the increasing of Δ/*λ* as shown in Fig. 10(b). The phenomenon can be understood as follows. From equation (19), we know 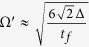, so the laser Ω′ increases with the increasing of detuning Δ. When Δ is large enough, the Zeno condition Ω′ ≪ *λ* for the non-resonant system is not ideally fulfilled, then the intermediate states including the cavity-excited states would be populated during the evolution, which would cause the system to be sensitive to the cavity decays. In other words, as long as the detuning Δ is small, the system is robust to the cavity decays as shown in Fig. 10. But substituting equation (19) into the condition Ω′ ≪ Δ, we deduce 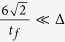, it denotes large Δ would be better. So, taking the two conditions into account, when the detuning Δ ≈ 1.5*λ*, atomic spontaneous emission and cavity field dissipation have an equal influence in the fidelity. According to the sensitivity of experimental apparatus to the atomic spontaneous emission and cavity field dissipation, we can reasonably select different parameters in practical. As we know, in general in order to restrain atomic spontaneous emission in QZD and cavity decay in STIRAP, we introduce detuning between the atomic transition, and that increases the evolution time. However in our scheme we only need to select appropriate parameter to restrain atomic spontaneous emission and cavity decay in a short time. To sum up, it is a better choice for the experimental researchers because the three-atom singlet state is generated much faster in the present shortcut scheme that contributes to the experimental research.

Finally, we present a brief discussion about the basic factors for the experimental realization of a three-atom singlet state. In a real experiment, the cesium atoms which have been cooled and trapped in a small optical cavity in the strong coupling regime^60^,^61^ can be used in this scheme. The state 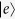 corresponds to *F* = 4, *m* = 3 hyperfine state of the 6^2^*P*_1/2_ electronic excited state, the state 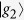 corresponds to *F* = 4, *m* = 3 hyperfine state of the 6^2^*S*_1/2_ electronic ground state, the state 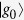 corresponds to *F* = 3, *m* = 2 hyperfine state of the 6^2^*S*_1/2_ electronic ground state, and the state 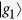 corresponds to *F* = 3, *m* = 4 hyperfine state of the 6^2^*S*_1/2_ electronic ground state, respectively. In recent experimental conditions^62^,^63^,^64^, it is predicted to achieve the parameters *λ* = 2*π* × 750 MHz, *κ* = 2*π* × 3.5 MHz, and *γ* = 2*π* × 2.62 MHz and the optical cavity mode wavelength in a range between 630 and 850 nm. By substituting the ratios *κ*/*λ* = 0.0047,*γ*/*λ* = 0.0035 into equation (21), we will obtain a high fidelity about 99.05%, which shows our scheme to prepare a three-atom singlet state is relatively robust. Nowadays, according to the literature^65^,^66^,^67^,^68^, the laser pulse which is used in our scheme can be obtained in a real experiment, so, our scheme is feasible in experiment.

## Summary

We have presented a promising method to construct shortcuts to adiabatic passage (STAP) for a three-atom system to generate singlet state in the cavity QED system. We simplify a multi-qubit system and choose the laser pulses to implement the fast generation of entangled states in light of quantum zeno dynamics and “transitionless quantum driving”. In comparison to QZD, the significant feature is that we do not need to control the evolution time exactly. As comparing with the STIRAP, the significant feature is the shorter evolution time. When dissipation is considered, we can find that the scheme is robust against the decoherence caused by both atomic spontaneous emission, photon leakage and operational imperfection. In addition, the present scheme only needs to select appropriate parameter to restrain atomic spontaneous emission and cavity decay in a short time. Numerical simulation result shows that the scheme has a high fidelity and may be possible to implement with the current experimental technology. In shorts, the scheme is robust, effective and fast. Actually, the present scheme in section III can be effectively applied to *N*-atom system for preparation of *N*-atom singlet state. We hope our work be useful for the experimental realization of quantum information in the near future.

## Additional Information

**How to cite this article**: Chen, Z. *et al.* Fast generation of three-atom singlet state by transitionless quantum driving. *Sci. Rep.* **6**, 22202; doi: 10.1038/srep22202 (2016).
